# Evaluation of a 4-week interdisciplinary primary care cardiovascular health programme: impact on knowledge, Mediterranean Diet adherence and biomarkers

**DOI:** 10.1136/bmjnph-2023-000790

**Published:** 2024-03-14

**Authors:** Lydia Tegwyn Mosher, Cindy Bizerra, Katelyn Davies, Jamie A Seabrook, Justine Keathley

**Affiliations:** 1 The East Elgin Family Health Team, Aylmer, Ontario, Canada; 2 School of Food and Nutritional Sciences, Brescia University College, The University of Western Ontario, London, Ontario, Canada; 3 Department of Paediatrics, The University of Western Ontario, London, Ontario, Canada; 4 Department of Epidemiology and Biostatistics, The University of Western Ontario, London, Ontario, Canada; 5 Children's Health Research Institute, London, Ontario, Canada; 6 Lawson Health Research Institute, London, Ontario, Canada; 7 Department of Human Health and Nutritional Sciences, University of Guelph, Guelph, Ontario, Canada

**Keywords:** Biomarker, Nutritional treatment, Preventive counselling

## Abstract

**Background:**

Cardiovascular disease (CVD) is the second-leading cause of death among Canadians. Clinical practice guidelines suggest that improvements to lifestyle, including dietary intake, can reduce the risk of CVD.

**Objectives:**

The primary aim of the study was to evaluate patient changes in adherence to the Mediterranean Diet (Medi-Diet) from baseline to 4-week and 6-month follow-up after participating in a 4-week, group-based, interdisciplinary cardiovascular health programme run by healthcare professionals (HCPs) in a primary care setting. Secondary outcomes included changes in blood pressure, total cholesterol, low-density lipoprotein-cholesterol, high-density lipoprotein cholesterol (HDL-c), triglycerides, non-HDL-c and haemoglobin A1c% from baseline to 6 months, and changes in knowledge scores from baseline to 4 weeks and 6 months. This study further aimed to compare outcomes between in-person programme delivery and virtual programme delivery during the COVID-19 pandemic.

**Methods:**

Participants (n=31) attended the Get Heart Smart (GHS) group-based educational and lifestyle behaviour change programme at the East Elgin Family Health Team for 4 weeks. Participants were 18 years or older and were referred by a HCP or self-referred to the GHS programme. Changes in the above-mentioned outcomes were evaluated. Due to the COVID-19 pandemic, the programme moved to a virtual mode of delivery, with 16 participants completing the programme in a virtual environment. Two-way repeated-measures analyses of variance were performed to explore if there were significant differences from baseline to 4-week and/or 6-month follow-up between groups (in-person compared with virtual) and within the pooled sample.

**Results:**

At baseline and 4-week follow-up, there were significant between-group differences in knowledge scores. After 6-month follow-up, there were statistically significant within-group improvements in Medi-Diet scores and knowledge scores in the pooled sample (n=31), in-person sample (n=15) and virtual sample (n=16). Apart from triglycerides, changes in biomarkers were all non-significant.

**Conclusions:**

The GHS programme effectively facilitated long-term (6-month) improved cardiovascular/lifestyle knowledge and adherence to the Medi-Diet. Transitioning to a virtual programme delivery did not impact the program’s ability to motivate nutrition-related behaviour change.

**WHAT IS ALREADY KNOWN ON THIS TOPIC**

- Improvements to lifestyle, such as following the Mediterranean Diet pattern, can optimise cardiovascular health.

**WHAT THIS STUDY ADDS**

- A 4-week educational and lifestyle behaviour change focused cardiovascular health programme (Get Heart Smart) led by dietitians, nurses and physicians resulted in improved Mediterranean Diet adherence.

**HOW THIS STUDY MIGHT AFFECT RESEARCH, PRACTICE OR POLICY**

- Adaptation of this programme among interdisciplinary care facilities could improve nutrition and health.

## Introduction

Approximately 1 in 12 Canadian adults live with diagnosed cardiovascular disease (CVD), and an estimated 14 Canadian adults, with diagnosed CVD, die every hour.^1 2^ Overall, CVD is the second-leading cause of death among Canadians.^3^ While rates of CVD are high, there are many known strategies used to help manage and reduce the risk of CVD. Current clinical practice guidelines suggest that improvements to lifestyle can optimise cardiovascular health through improving lipid profile and blood pressure (BP).^4^ Dyslipidaemias can contribute to atherosclerosis and subsequently CVD.^5^ Moreover, elevated BP has been associated with heart failure, stroke and heart attack.^6^ In addition, higher levels of haemoglobin A1c (HbA1c) have also been strongly associated with a high risk of CVD in people with and without diabetes mellitus.^7 8^

Lifestyle improvements that can optimise cardiovascular health include limiting alcohol, reducing stress, being physically active, weight management, quitting (or not starting) smoking and maintaining a balanced and nutrient-dense dietary pattern.^9^ A specific dietary pattern that has been consistently associated with numerous health benefits, including a reduced risk of metabolic diseases, neurodegenerative diseases, cancer and CVD, is the Mediterranean Diet (Medi-Diet).^10^ The Medi-Diet is a dietary pattern that encourages high intakes of unsaturated fat sources, leafy green vegetables, fruits, whole-grain cereals, nuts, seeds and plant-based proteins such as pulses and legumes; a moderate intake of animal proteins including fish, meat and dairy products; and a more limited intake of sweets.^11^

Many studies have found that cardiovascular health interventions, including those that emphasise patient education and goal setting, have been effective in improving cardiovascular outcomes.^12 13^ Theoretical foundations can also guide health programme design. For example, in Ajzen’s Theory of Planned Behaviour (1985), knowledge is a significant indicator of engagement as it impacts all three intentions within the Theory of Planned Behaviour: attitudes, subjective norms and perceived behavioural control.^14 15^

The purpose of the study was to determine if 4 weekly educational and lifestyle behaviour change focused sessions related to cardiovascular health (The Get Heart Smart (GHS) programme) helps to improve adherence to the Medi-Diet. The secondary aim of the study was to determine if changes in BP, HbA1c, lipid profiles and an understanding of cardiovascular health occurred after a 4-week and a 6-month follow-up. Lipid profiles include total-cholesterol (total-c), low-density lipoprotein-cholesterol (LDL-c), high-density lipoprotein cholesterol (HDL)-c, non-HDL-c and triglycerides (TG). In addition, due to the COVID-19 pandemic, the study also evaluated the feasibility of moving the GHS programme to a virtual environment, while further comparing the above-mentioned outcomes between participants who completed the programme virtually versus those who completed it in-person.

## Methods

### Study design

This was a pragmatic, longitudinal cohort study, which evaluated a primary care group-based lifestyle programme, with a pragmatic delivery (ie, an investigation focused on practical, real-world delivery), centered on cardiovascular health. Convenience sampling was used to recruit participants via referrals from healthcare professionals and self-referrals at the East Elgin Family Health Team.

### Study population

Any patients who were referred to the GHS programme by their physician, nurse practitioner, registered nurse or dietitian or who self-referred were invited to participate in the study provided they met the inclusion criteria of being over 18 years of age. There were no further exclusion criteria as this was a pragmatic study that aimed to evaluate the effectiveness of a public health programme in which any adult from the general public was eligible to participate in. As such, the present study aimed to evaluate data from all participants eligible to participate in the programme. Data were collected from May 2019 to March 2023. After referral to the GHS programme and invitation to participate in the study, informed consent was obtained. Due to the COVID-19 pandemic, in March 2020 and onwards, consent was obtained virtually, with study investigators reviewing study information with participants over the phone and participants signing and returning consent forms via the OCEAN platform, V.4.29.

### GHS programme

The GHS programme is a standardised programme, which was designed by registered dietitians at the East Elgin Family Health Team (Aylmer, Ontario, Canada) and consisted of 4 weekly educational sessions that each lasted for approximately 75 min. Each session provided evidence-based health information and advice on a specific topic, as follows: (1) Introduction to heart disease and BP management, which provided an introduction to heart disease and BP, discussed nutrition and lifestyle measures that impact BP, and provided recommendations to manage BP including a reduction in sodium, alcohol, and caffeine intake, smoking cessation, increasing physical activity and potassium intake, and stress management; (2) Cholesterol management, which reviewed blood lipids and targets, dietary and lifestyle measures that impact cholesterol, and recommendations to manage cholesterol, namely limiting intake of saturated and trans fats and opting for unsaturated fat sources instead, reducing alcohol and sugar intake, smoking cessation, and increasing fibre intake and physical activity levels; (3) Heart-Health-Focused Grocery Store Tour, where session instructors taught participants how to navigate through a grocery store from a heart-health perspective and learnt practical tools such as nutrition facts table reading and (4) Dietary Patterns for Optimal Heart Health, which reviewed dietary patterns that have been shown in the literature to be beneficial for heart health, including the Dietary Approaches to Stopping Hypertension (DASH) diet, the Medi-Diet and the Portfolio Diet.^16–21^ During one of the four sessions, participants were joined by a physician and had a question-answer period pertaining to medications for heart health. Additionally, at the end of each education session, participants had time to create 1 SMART (specific, measurable, achievable, realistic and timely) goal pertaining to the information that was covered during that session. Participants were invited to discuss their progress with their SMART goal at the beginning of the following session, although this was not mandatory. Educational sessions initially took place in person (May 2019–March 2020) and were facilitated primarily by two registered dietitians or a registered dietitian and registered nurse, with cofacilitation by a physician who participated in a question-and-answer period during one of the sessions about medications. Handouts relevant to the educational topic were also provided to participants in person. In session 1, participants were provided with handouts on hypertension (adapted from ‘Healthy Eating for BP'’ by Hypertension Canada, 2015), Using the Nutrition Facts Table: % Daily Value (developed by Health Canada, 2011) and a list of local resources to help manage stress, anxiety, depression and happiness. In session 2, handouts on how to improve an individual’s fat and soluble fibre intake (both developed by Niagara Regional Dietitians—Heart Healthy Committee, 2013) were provided. In session 3, a handout entitled Learning about Sugars Labelling: New Nutrition Information (developed by the Canadian Sugar Institute, 2019) was provided. In session 4, participants were provided with handouts entitled: Healthy Eating to Lower Your BP—Reducing Sodium and DASH (developed by the Heart and Stroke Foundation), Eating the Mediterranean Way for Good Health and a Longer Life (developed by VA Health Care, 2015), Medi-Diet and Mediterranean Plate (developed by University Hospital Network, 2017) and The Portfolio Diet (developed by Hamilton Health Sciences, 2013).

In March 2020, given the COVID-19 pandemic, the educational sessions and distributions of handouts transitioned to a virtual model, taking place over the Ontario Telemedicine Network platform, and being delivered to participants through the secure email platform, OCEAN, software V.4.29, respectively. During the forced transition of the GHS programme to a virtual model, the slide decks, handouts and standardised delivery of the GHS programme remained the same. However, session 3 (Heart-Health-Focused Grocery Store Tour) transitioned to a virtual presentation presented through slide decks, as physical in-person group grocery store tours were not feasible. Grocery Store Tour slide decks reviewed each department (produce, bakery, fish and seafood, meat, dairy and eggs) and continued to review practical tools such as nutrition facts table reading. Overall, the only change to the programme delivery was the virtual delivery as opposed to in-person delivery.

### Primary and secondary outcomes

The primary outcome of the study was to evaluate the change in participants’ adherence to the Medi-Diet from baseline to 6-month follow-up. Secondary outcomes included change in participants’ BP and knowledge questionnaire from baseline to 4-week follow-up and baseline to 6-month follow-up, change in participants’ lipid profiles and HbA1c from baseline to 6-month follow-up and change in Medi-Diet scores after 4-week follow-up. Due to the COVID-19 pandemic, BP was only collected for the in-person facilitation of the GHS programme, as it could not be completed virtually.

### Data collection

Data were collected at three time points: baseline, at the end of the 4-week GHS programme and at 6-month follow-up. Initially, data were collected in person, however, in March 2020, data collection transitioned to a virtual model due to the COVID-19 pandemic.

At baseline, demographic information was collected including participant age, sex and ethnicity. Other baseline data included the GHS knowledge questionnaire (online supplemental file 1), Medi-Diet Score Tool, systolic BP (SBP) and diastolic BP (DBP) measured using the Welch Allyn Reusable ProBP 2400 BP Cuff, as well as lipid profiles (total-c, LDL-c, HDL-c, non-HDL-c, TG) and HbA1c. Lipid profiles and HbA1c were measured via blood draw. Laboratory requisitions were obtained from the participant’s primary care physician and participants were instructed to visit their local medical laboratory service to fulfil the requisition through blood draw. Lipids were evaluated using photometric and potentiometric methodology to measure metabolites on the Abbott Alinity c-series platform. HbA1c was evaluated via the turbidimetric inhibition immunoassay for haemolysed whole blood on the Roche c513 platform. The GHS knowledge questionnaire was developed by CB and JK and was based on a previously validated and reliable questionnaire, established by England’s National Health Service used to evaluate patients’ cardiovascular health knowledge and risk perceptions.^22^ The adapted questionnaire consisted of nine questions pertaining to dietary and lifestyle measures and their implications on heart health. The Medi-Diet score was obtained via the 14-question validated Medi-Diet Score Tool.^23^

10.1136/bmjnph-2023-000790.supp1 Supplementary data

At the end of the 4-week GHS programme, data collection included knowledge scores, Medi-Diet Scores and GHS attendance for all participants. SBP and DBP were also measured for the in-person GHS participants.

Six months after the completion of the GHS programme, data collection included GHS knowledge scores, Medi-Diet scores, lipid profiles, HbA1c and medication changes relevant to BP and/or lipids for all participants. SBP and DBP were also measured for the in-person GHS participants.

### Sample size calculation

The primary outcome of interest was a change in Medi-Diet Scores from baseline to 6-month follow-up. Using a sample size calculation with an alpha of 0.05 and with 95% power to detect an effect size of 1.4 points for change in the Medi-Diet score (as reported by Brauer *et al*), while accounting for a standard 20% drop-out, at least 12 participants (per group) were needed to detect a statistically significant change from baseline to 6-month follow-up.^24^

### Statistical analyses

Statistical analyses were conducted using SPSS Statistics V.29.0.1.0 (IBM). The mean and SD were used to report continuous variables and percentages were used to summarise categorical variables. Data analysis compared within-group changes for both the in-person GHS sample and virtual GHS sample independently and pooled, as well as between-group changes of the in-person compared with virtual GHS samples. Differences in baseline variables between the in-person and the virtual group were compared using independent t-tests for continuous variables and χ^2^ tests for categorical variables. Two-way repeated-measures analysis of variance (RM-ANOVAs) was performed to explore if there were significant differences from baseline to 4-week and 6-month follow-up between and within groups as the Medi-Diet and knowledge scores, both dependent variables, were measured at three time points. Visual inspections via histograms were used to assess normality, and given minimal departure from normality, the two-way RM-ANOVA was used for consistency throughout.

## Results

### Participant characteristics

The target sample size was achieved with a total of 37 participants enrolling in this study, with 6 participants dropping out by the 6-month time point (participant retention rate: 83.8%). Complete-case analysis (listwise deletion) was used to handle the missing data given the small number of dropouts.^25^ A total of 15 participants completed the study in person, while 16 participants completed the study virtually (due to the mandatory switch to a virtual environment during the COVID-19 pandemic). Participants’ baseline characteristics are detailed in table 1. The sample consisted of primarily female, Caucasian adults with a mean age of 61.3 years±8.1 with overall healthy lipid profiles (table 1). The sample was generally healthy based on their baseline serum lipid, BP and HbA1c levels (table 1 and online supplemental 1). Lipid levels were considered healthy/normal if they were as follows: cholesterol between 3.5 mmol/L and 5.2 mmol/L, HDL-c greater than 1.3 mmol/L (females) and 1.0 mmol/L (males), LDL-c below 3.0 mmol/L, non-HDL below 4.5 mmol/L, TG below 1.7 mmol/L and HbA1c below 6%.^26 27^

**Table 1:** Baseline demographics and clinical characteristics of participants

Baseline characteristics	Pooled sample (n=37)		In-person group (n=20)		Virtual group (n=17)		P value
	Frequency	Per cent	Frequency	Per cent	Frequency	Per cent	
Gender	27/37	73.0% Female	15/20	75.0% Female	12/17	70.6% Female	0.76
Ethnicity	100/100	100% Caucasian	100/100	100% Caucasian	100/100	100% Caucasian	N/A*

	Mean±SD	Mean±SD	Mean±SD	
Age	61.3±8.1	59.5±8.5	63.4±7.3	0.61
Total cholesterol (mmol/L)	4.7±1.1	4.4±1.1	5.0±1.0	0.80
LDL-cholesterol (mmol/L)	2.5±1.0	2.3±1.0	2.7±0.9	0.93
HDL-cholesterol (mmol/L)	1.5±0.4	1.5±0.5	1.4±0.4	0.47
HbA1c (%)	6.0±0.6	6.0±0.6	6.0±0.7	0.82
TG (mmol/L)	1.7±0.9	1.5±0.7	2.0±1.0	**0.046**
Non-HDL-cholesterol^3^ (mmol/L)	3.6±1.2	2.9±1.1	3.6±1.2	0.73
Participant attendance	3.5±0.7	3.4±0.8	3.6±0.5	0.28

Systolic and diastolic BP means, SD and p values are not reported as it was not possible to complete BP data collection with the mandatory transition to a virtual environment.

Bold values are significant at p<0.05.

*Ethnicity p value not applicable as all subjects were Caucasian.

BP, blood pressure; HbA1c, haemoglobin A1c; HDL, high-density lipoprotein; LDL, low-density lipoprotein; N/A, not available.

Attendance in the GHS programme was high, with participants attending a mean of 3.5 out of 4 sessions; there were no significant differences in attendance between the in-person and virtual groups (table 1).

### Medi-Diet scores

**Figure 1:**
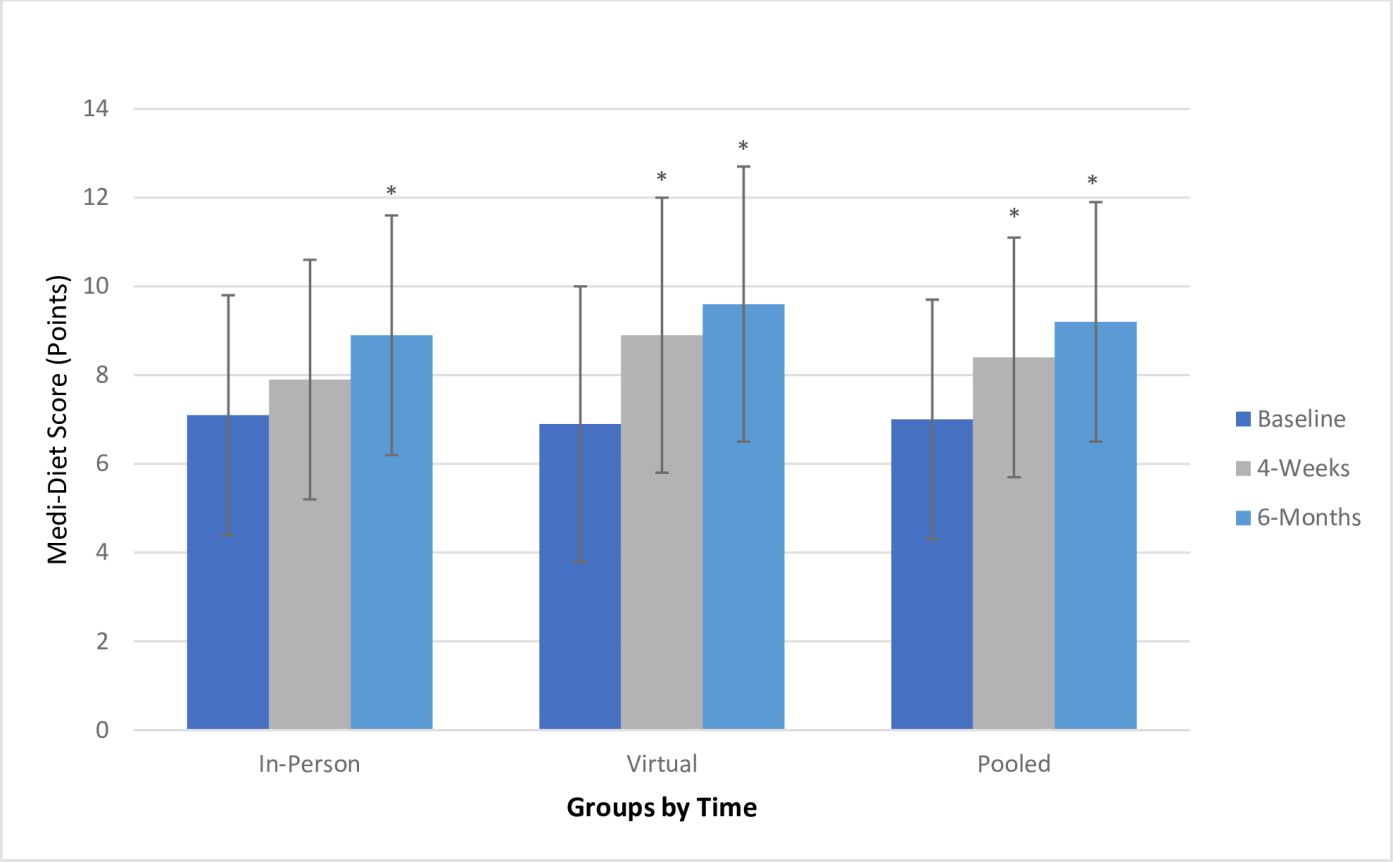
Change in Medi-Diet Score after 4-week and 6-month follow-up. Total (pooled) sample: n=31 (n=15 in-person, n=16 virtual). In-person (mean±SD): baseline=7.1±2.7, 4 weeks=7.9±3.1, 6 months=8.9±2.7. Virtual (mean±SD): baseline=6.9±2.3, 4 weeks=8.9±1.8, 6 months=9.6±1.6. Pooled (mean±SD): baseline=7.0±2.4, 4 weeks=8.4±2.5, 6 months=9.2±2.2. *Statistically significant at p<0.05. *Within-group differences. In-person: p value at baseline to 4 weeks (p=0.317) baseline to 6 months (p=0.012). Virtual: p value at baseline to 4 weeks (p<0.001), baseline to 6 months (p<0.001). Pooled sample: p value at baseline to 4 weeks (p<0.001), baseline to 6 months (p<0.001). Between-group differences: baseline (p=0.828); 4 weeks (p=0.279); 6 months (p=0.430). Partial eta squared: time 0.396 (large); time×group 0.048 (small). Medi-Diet, Mediterranean Diet.

Results from the analyses of baseline, 4-week and 6-month Medi-Diet scores (primary outcome) are shown in figure 1. At baseline, there was no statistically significant difference in Medi-Diet Scores between the in-person and virtual groups. Immediately following the 4-week GHS programme, there were significant improvements in adherence to the Medi-Diet within the virtual group. After 6-month follow-up, there were significant improvements in adherence to the Medi-Diet in both the in-person group and the virtual group, as well as the pooled sample. As time increased, there was a large magnitude of effect on Medi-Diet adherence (partial eta squared for time: 0.396).

### Knowledge questionnaire scores

**Figure 2:**
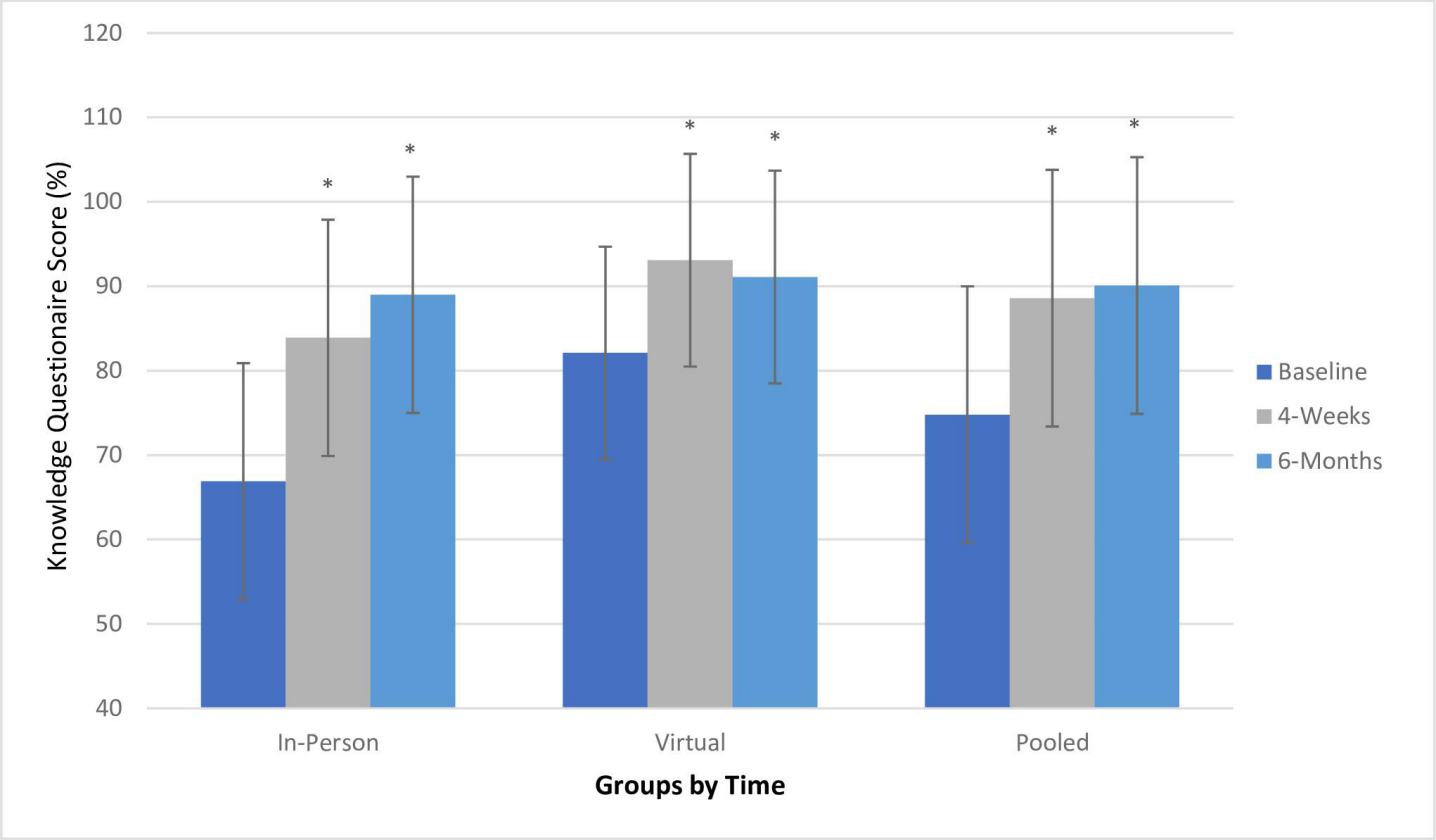
Change in Knowledge Questionnaire Scores after 4-week and 6-month Follow-Up. Total (pooled) sample: n=31 (n=15 in-person, n=16 virtual). In-person (mean±SD): baseline=66.9±14.0, 4 weeks=83.9±10.9, 6 months=89.0±12.5. Virtual (mean±SD): baseline=82.1±12.6, 4 weeks=93.1±8.9, 6 months=91.1±12.8. Pooled (mean±SD): baseline=74.8±15.2, 4 weeks=88.6±10.8, 6 months=90.1±12.5. *Statistically significant at p<0.05. *Within-group differences. In-person: p value at baseline to 4 weeks (p<0.001), baseline to 6 months (p<0.001) Virtual: p value at baseline to 4 weeks (p= 0.015), baseline to 6 months (p=0.014). Pooled sample: p value at baseline to 4 weeks (p<0.001), baseline to 6 months (p<0.001). Between-group differences: baseline (p=0.004); 4 weeks (p=0.014); 6 months (p=0.654). Partial eta squared: time 0.476 (large); time×group 0.119 (moderate).

Results from the analyses of baseline, 4-week and 6-month knowledge questionnaire scores are shown in figure 2. At baseline, there was a significant difference in knowledge questionnaire scores between the in-person and virtual groups. In all groups (virtual, in-person and pooled), there were significantly improved knowledge scores after 4-week and 6-month follow-ups compared with baseline. At the 4-week follow-up, knowledge levels between the in-person and virtual groups were significantly different, however at the 6-month follow-up, no significant difference was found between groups. As time increased, there was a large magnitude of effect on knowledge score (partial eta squared for time: 0.476).

### Biomarkers

Results from the analyses of baseline, 4-week and 6-month total cholesterol, LDL-c, HDL-c, HbA1c%, TG and non-HDL-c participant biomarkers are shown in online supplemental table 2. The results of the analyses were all non-significant for biomarkers apart from TG levels at baseline between groups, as shown in table 1 (p=0.046).

## Discussion

These findings suggest that after participating in a 4-week, group-based cardiovascular health programme, adults significantly improved their adherence to the Medi-Diet. The findings of our study are generalisable primarily to older adult females given the demographics of the participants who took part in the study. Although the educational sessions were standardised across in-person and virtual groups, our finding of 4-week improvements in Medi-Diet scores in the virtual group only was intriguing. It is possible that motivation to engage in short-term dietary change was greatest in those participating in the virtual programme. The virtual setting may have allowed participants to connect deeper to their engagement and thus increased their behavioural change responses. This insight into the potential benefit of virtual nutrition education can help guide future knowledge dissemination techniques.

While our findings of improved Medi-Diet adherence in the GHS programme were statistically significant, it is important to note that they were also clinically meaningful. Previous studies support an inverse association between adherence to the Medi-Diet pattern and risk of CVD.^28–33^ In a study assessing the relationship between Medi-Diet and risk of stroke, a greater adherence to the Medi-Diet was associated with lower risk of incident coronary heart disease and stroke in females.^34^ In the present study, the average Medi-Diet score increased from 7.0 at baseline to 9.2 at the 6-month follow-up. This 2.2-point increase is statistically significant and has been shown to translate to improvements in health outcomes. For example, a 2-point increase in Medi-Diet score has been associated with improved health status, including an 8% reduction in overall mortality, 10% reduced risk of CVD, 4% reduced risk of neoplastic disease and 32% reduced risk of depression.^33 35^ In addition, Stefler *et al* reported that a 2.2-point increase in Medi-Diet Score was inversely associated with all cause-mortality and CVD.^36^ From a metabolic standpoint, these health outcomes relate to the Medi-Diet’s anti-inflammatory and antioxidative capabilities, as well as regulatory effect on fatty acids.^23 28 29^

Many dietary strategies have been demonstrated to influence cardiovascular health outcomes. While a low-fat diet, of which less than 30% of calories come from fat, is an alternative diet that has been demonstrated to improve cardiovascular health,^30^ adherence to the Medi-Diet has been shown to be superior in reducing the rate of major cardiovascular events.^30^ The DASH diet has also been demonstrated to improve cardiovascular health, more specifically BP.^37^ However, research has demonstrated that the Medi-Diet, when compared with a DASH diet, was associated with lower risk of 10-year fatal and non-fatal CVD.^37^ As such, it has been suggested that public health interventions focus on enhancing adherence to the Medi-Diet.^37^ Within Canada, adaptability of the Medi-Diet is possible as it encourages a dietary intake of fruits, vegetables, whole grains and plant-based proteins, all foods locally available to Canadian residents. Of note, personalised interventions and available resources should be considered when choosing the appropriate diet, as both impact long-term diet adherence.^38 39^

In addition to our main outcome of improved Medi-Diet adherence, we also demonstrated significant improvements in knowledge scores in the in-person, virtual and pooled samples, with moderate-to-large effect sizes. This finding is directly related to our findings of improved Medi-Diet adherence, in considering established and validated behaviour change theory. For example, the Theory of Planned Behaviour states that knowledge is a significant indicator of engagement, which can in turn influence intentions and resulting behaviour change.^14 15^

Despite the improvements in Medi-Diet adherence and knowledge scores, there were no significant changes in the lipid profiles of patients from baseline to 6 months. Patients’ baseline cardiovascular biomarkers were overall within normal limits and thus participants of this study were already in good cardiovascular health at baseline, which may help explain why there were no significant changes in biomarkers at 6-month follow-up.

As an exploratory outcome due to the mandatory switch to a virtual environment during the COVID-19 pandemic, this study further aimed to compare outcomes between in person programme delivery and virtual programme delivery. The present study transitioning to a virtual platform did not appear to have a significant impact on the final 6-month follow-up Medi-Diet and knowledge score between the in-person and virtual groups. Therefore, the transition did not appear to impact the programme’s ability to motivate improved knowledge and nutrition-related behaviour change in person or virtually by the 6-month follow-up. GHS programme attendance also did not differ in an in-person environment compared with a virtual environment. These findings relate to a similar study which evaluated a cardiovascular health improvement programme for Latinos during the COVID-19 pandemic and found that participants were accepting of the virtual programme delivery.^40^

While the strengths of this study include a pragmatic programme delivery in a primary care setting, a sample size adequately powered to detect significant differences in the primary outcome (change in Medi-Diet scores), among others, there were some limitations. First, given the lack of a control group, it is possible that confounding factors that were not accounted for in this study could have influenced the results. Second, the sample consisted primarily of females, resulting in an under-representation of the male population. Additionally, there was a lack of ethnic diversity present as the sample consisted of exclusively Caucasian individuals. These factors impact the studies’ generalisability to the greater population as they focuse on a specific population. Future research should aim to validate these findings in a more diverse population to improve generalisability. Another limitation, consistent with other studies that were ongoing during the pandemic, includes the added barriers of the pandemic and the impact of this on health behaviour change,^39–41^ which was not evaluated in the present study. The pandemic had a significant negative impact on the population’s daily lifestyle habits, including eating patterns and physical activity, which could have impacted the study’s findings.^42 43^ Given these findings, it is possible that a virtual programme delivery could lead to greater dietary changes compared with the in-person programme, but we were unable to evaluate this due to the absence of control groups. Last, while the sample size was large enough to detect differences in our primary outcome, it is possible that this study was underpowered for detecting significant differences in the biomarkers evaluated. However, it is also important to reiterate that the study population was generally healthy as baseline (in terms of the biomarkers evaluated in the present study); repeating this study in a population with poor baseline cardiovascular health may demonstrate different findings. Future research should evaluate the GHS programme in different populations, such as those with CVD.

Overall, the results of this study provide insight into the beneficial impact of participating in just 4 weekly cardiovascular education sessions led by a team of dietitians, nurses and physicians on nutrition-related behaviour change. The GHS programme should be adapted and evaluated by other primary care and public health facilities, while focusing on recruiting a larger, more diverse sample of participants.

## Data Availability

Data are available on reasonable request. Not applicable.
