# Supplementary material for: Evaluation of a 4-week interdisciplinary primary care cardiovascular health programme: impact on knowledge, Mediterranean Diet adherence and biomarkers
Source: BMJ Nutr Prev Health. 2024 Mar 14;7(1):95–102. doi: 10.1136/bmjnph-2023-000790 (PMC11221286; doi:10.1136/bmjnph-2023-000790)
Supplement: Supplementary data [file bmjnph-2023-000790supp001.pdf]

Supplementary Table 1: Systolic (SBP) and Diastolic Blood Pressure (DBP) for the In-Person Group

	**SBP**		**DBP**	
	Mean±SD (n=4)	Mean±SD (n=19)	Mean±SD (n=4)	Mean±SD (n=19)
**Baseline**	128.8±10.2	129.8±16.9	73.0±6.5	72.9±8.0
**4-week follow-up**	131.5±10.5	131.6±15.1	74.3±9.8	76.5±12.5
**6-month follow-up**	125.8±11.6	N/A	74.8±15.6	N/A

Within-group differences
SBP (n=4): *p*-value at BL to 4w (*p*=0.105), BL to 6m (*p=0.308),* 4w to 6m (*p=0*.176)
SBP (n=19): *p*-value at BL to 4w (*p*=0.461)
DBP (n=4): *p*-value at BL to 4w (*p*=1.000), BL to 6m (*p*=1.000*),* 4w to 6m (*p*=1.000)
DBP (n=19): *p*-value at BL to 4w (*p*=0.056)

Supplementary Table 2: Biomarkers for the in-person, virtual, and pooled samples at baseline and 6-month follow-up

	**In-Person Group (n=15)**			**Virtual Group (n=16)**			**Pooled Sample (n=31)**			**Between-Group Differences**		**Partial Eta Squared[7]**	
**Variable**	Baseline (Mean ± SD)	6 months (Mean ± SD)	*p*-value[1]	Baseline (Mean ± SD)	6 months (Mean ± SD)	*p*-value[2]	Baseline (Mean ± SD)	6 months (Mean ± SD)	*p*-value[3]	*p*-value[4] Baseline	*p*-value[4] 6 months	Time	Time*Group
Total Cholesterol[5]	4.6±1.1	4.7±0.9	0.57	4.9±1.0	4.8±0.9	0.43	4.8±1.0	4.8±0.9	0.94	0.478	0.901	N/A	0.035 (small)
LDL-Cholesterol (mmol/L)[5]	2.5±1.0	2.6±0.7	0.20	2.7±0.9	2.5±0.8	0.17	2.6±0.9	2.5±0.7	0.73	0.544	0.881	0.005 (small)	0.043 (small)
HDL-Cholesterol (mmol/L)[5]	1.5±0.4	1.5±0.3	0.74	1.4±0.3	1.4±0.3	0.77	1.4±0.4	1.4±0.3	0.66	0.500	0.438	0.008 (small)	0.000 (N/A)
HbA1c (%)[6]	5.8±0.5	5.9±1.0	0.19	6.0±0.6	6.0±0.6	0.70	5.9±0.6	6.0±0.8	0.07	0.288	0.828	0.022 (small)	0.060 (moderate)
TG (mmol/L)[5]	1.5±0.8	1.6±1.0	0.69	1.9±0.9	2.0±1.1	0.54	1.7±0.9	1.8±1.1	0.48	0.299	0.351	0.020 (small)	0.000 (N/A)
Non-HDL-Cholesterol(mmol/L)[5]	3.1±1.0	3.3±0.9	0.49	3.5±1.1	3.4±1.0	0.44	3.4±1.1	3.4±0.9	0.97	0.351	0.717	N/A	0.041 (small)

HbA1c: hemoglobin A1c, HDL: High-Density Lipoprotein, LDL: Low-Density Lipoprotein,

1. *p*-values for within-group (in-person) changes between baseline to 6 months
2. *p*-values for within-group (virtual) changes between baseline to 6 months
3. *p*-values for within-group (pooled sample) changes between baseline to 6 months
4. *p*-values for between group (in-person vs. virtual) differences between baseline to 6 months
5. n= 27 (11 in-person, 16 virtual)
6. n= 28 (12 in-person, 16 virtual)
7. Interpreted according to Cohen (1988), a partial eta squared of 0.01 is small, 0.06 is moderate, and 0.13 or more is a large effect size.
